# Supplementary material for: Multiple evolutionary origins and losses of tooth complexity in squamates
Source: Nat Commun. 2021 Oct 14;12:6001. doi: 10.1038/s41467-021-26285-w (PMC8516937; doi:10.1038/s41467-021-26285-w)
Supplement: Supplementary file 1 — Supplementary Information [file 41467_2021_26285_MOESM1_ESM.pdf]

Supplementary Information for

Multiple evolutionary origins and losses of tooth complexity in squamates

F. Lafuma, I. J. Corfe, J. Clavel, N. Di-Poï

This study is supported by the following supplementary information:

Supplementary Figures: includes Supplementary Figures 1–12 with details of the super-tree nomenclature, geometric morphometric analyses, ancestral character state reconstructions, and models of diversification.

Supplementary Tables: includes Supplementary Tables 1–14, covering details of geometric morphometric analyses, ancestral character state reconstructions, tests of correlated evolution, rates of phenotypic evolution, and diversification models.

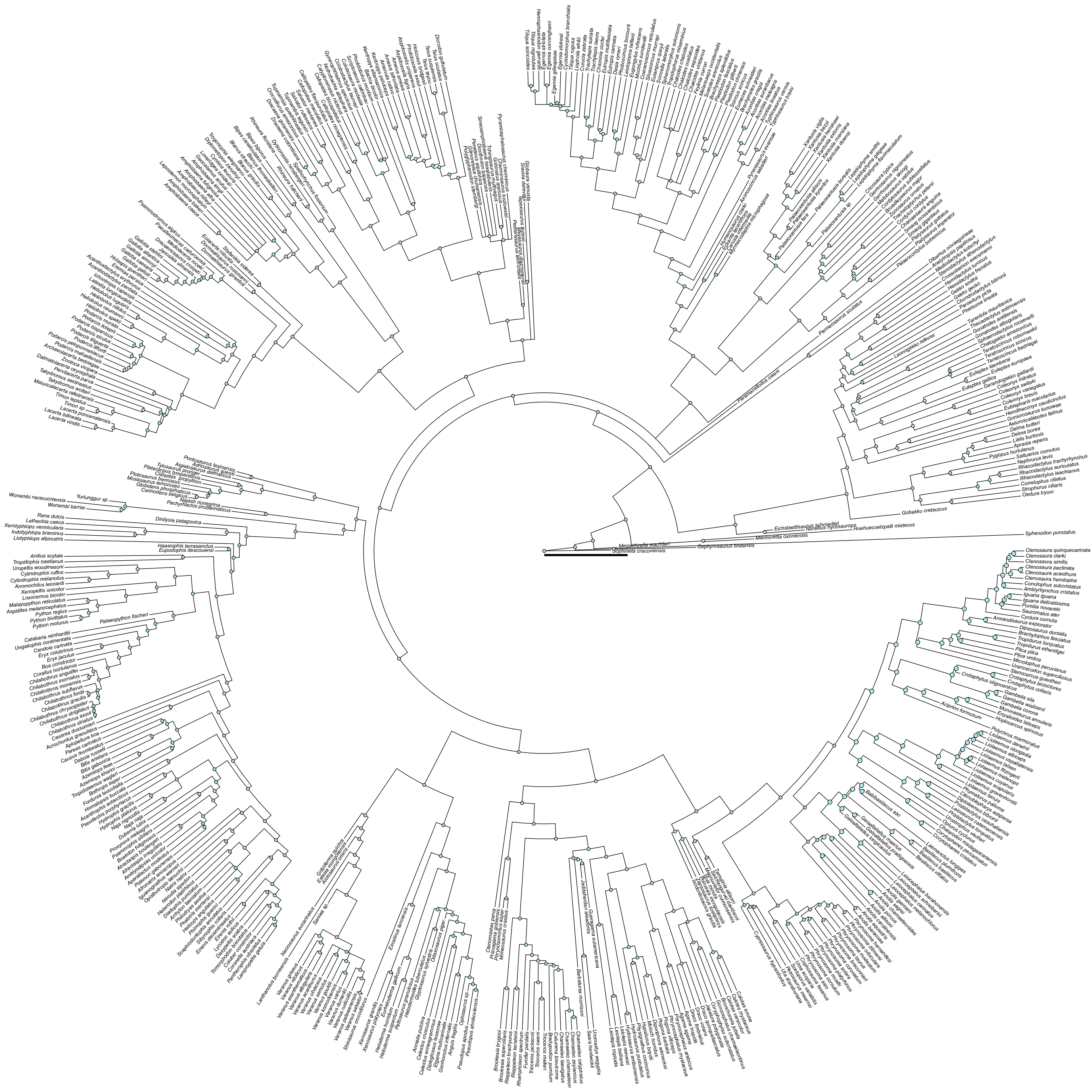

Supplementary Figures

Supplementary Fig. 1 | Time-calibrated polytomous squamate phylogeny with node nomenclature. Informal super-tree including 545 extant and extinct squamates species and three outgroup species (see Methods). Labels indicate node numbers as referred in the text and Supplementary Information. Scalebar = 50 million years.

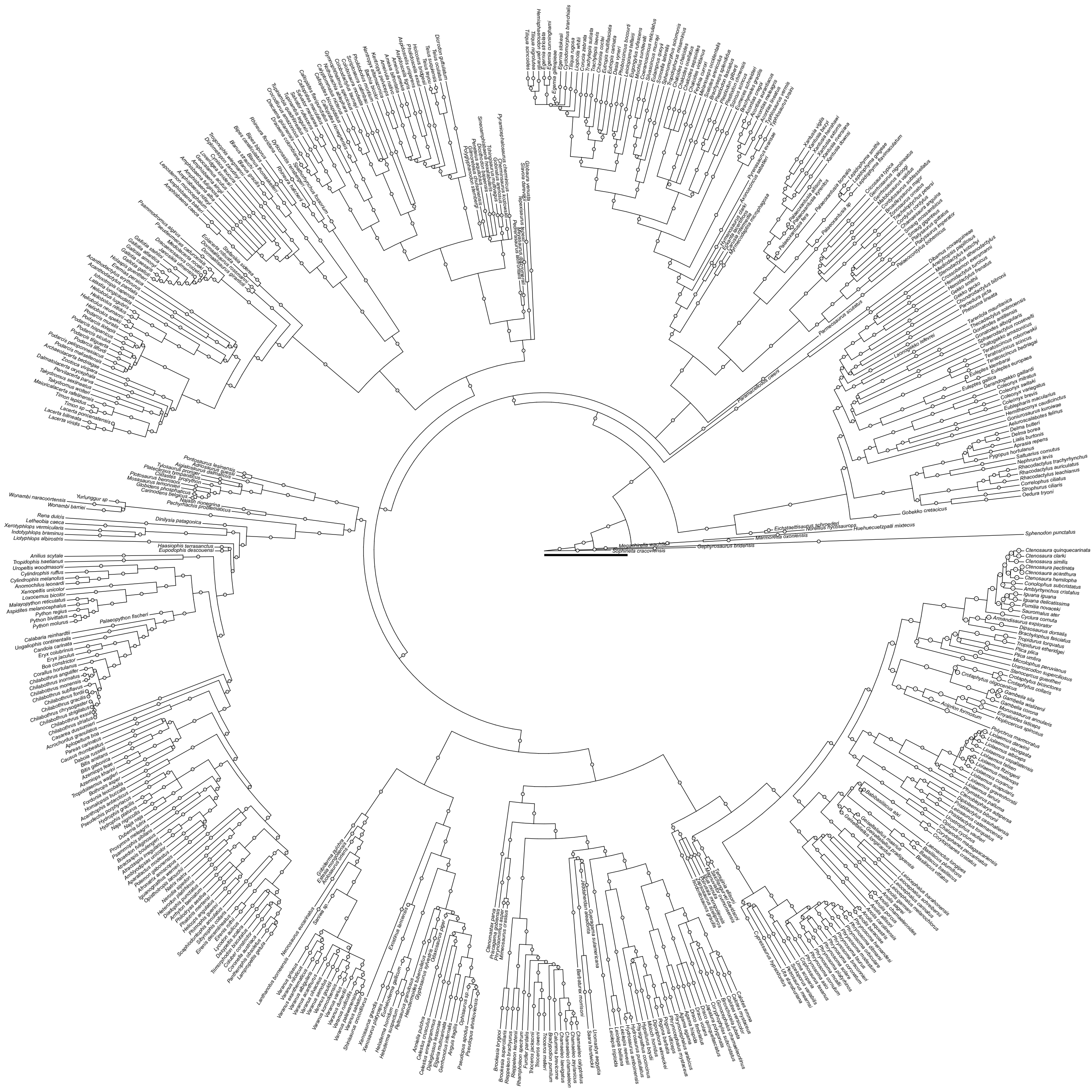
Sphenodon punctatus
Gephyrosaurus bridensis
Marmoretta oxoniensis
Eichstaettisaurus schroederi
Norellius nyctisaurops
Huehuecuetzpalli mixtecus
Megachirella wachtleri
Sophineta cracoviensis
Gobekko cretacicus
Oedura tryoni
Strophurus ciliaris
Correlophus ciliatus
Rhacodactylus leachianus
Rhacodactylus auriculatus
Rhacodactylus trachyrhynchus
Nephrurus levis
Saltuarius cornutus
Pygopus hortulanus
Aprasia repens
Lialis burtonis
Delma borea
Delma butleri
Aeluroscalabotes felinus
Goniurosaurus kuroiwae
Hemitheconyx caudicinctus
Eublepharis macularius
Coleonyx brevis
Coleonyx variegatus
Coleonyx switaki
Coleonyx mitratus
Gerandogekko gaillardi
Eublepharis macularius
Ctenosaura quinquecarinata
Ctenosaura clarki
Ctenosaura similis
Ctenosaura pectinata
Ctenosaura acanthura
Ctenosaura hemilopha
Conolophus subcristatus
Amblyrhynchus cristatus
Iguana iguana
Iguana delicatissima
Pumilia novaceki
Sauromalus ater
Cyclura cornuta
Armandisaurus explorator
Dipsosaurus dorsalis
Brachylophus fasciatus
Tropidurus torquatus
Tropidurus etheridgei
Plica plica
Plica umbra
Microlophus peruvianus
Uranoscodon superciliosus
Stenocercus guentheri
Crotaphytus bicinctores
Crotaphytus collaris
Gambelia sila
Gambelia wislizenii
Gambelia corona
Morunasaurus annularis
Enyalioides laticeps
Hoplocercus spinosus
Aciprion formosum
Polychrus marmoratus
Pontosaurus lesinensis
Adriosaurus suessi
Aigialosaurus dalmaticus
Tylosaurus proriger
Platecarpus tympaniticus
Plotosaurus bennisoni
Clidastes propython
Mosasaurus lemonnieri
Globidens phosphaticus
Carinodens belgicus
Najash rionegrina
Pachyrhachis problematicus
Wonambi naracoortensis
Yurlunggur sp
Wonambi barriei
Rena dulcis
Letheobia caeca
Xerotyphlops vermicularis
Indotyphlops braminus
Liotyphlops albirostris
Dinilysia patagonica
Haasiophis terrasanctus
Eupodophis descouensi
Anilius scytale
Tropidophis haetianus
Uropeltis woodmasoni
Cylindrophis ruffus
Cylindrophis melanotus
Anomochilus leonardi
Xenopeltis unicolor
Loxocemus bicolor
Malayopython reticulatus
Aspidites melanocephalus
Python regius
Python bivittatus
Python molurus
Palaeopython fischeri
Calabaria reinhardtii
Ungaliophis continentalis
Candoia carinata
Eryx colubrinus
Eryx jaculus
Boa constrictor
Corallus hortulanus
Chilabothrus angulifer
Chilabothrus inornatus
Chilabothrus monensis
Chilabothrus subflavus
Chilabothrus fordii
Chilabothrus gracilis
Chilabothrus chrysogaster
Chilabothrus strigilatus
Chilabothrus exsul
Chilabothrus striatus
Casarea dussumieri

Supplementary Fig. 2 | Time-calibrated polytomous squamate phylogeny with branch nomenclature. Informal super-tree including 545 extant and extinct squamates species and three outgroup species (see Methods). Labels indicate branch numbers as referred in the text and Supplementary Information. Scalebar = 50 million years.

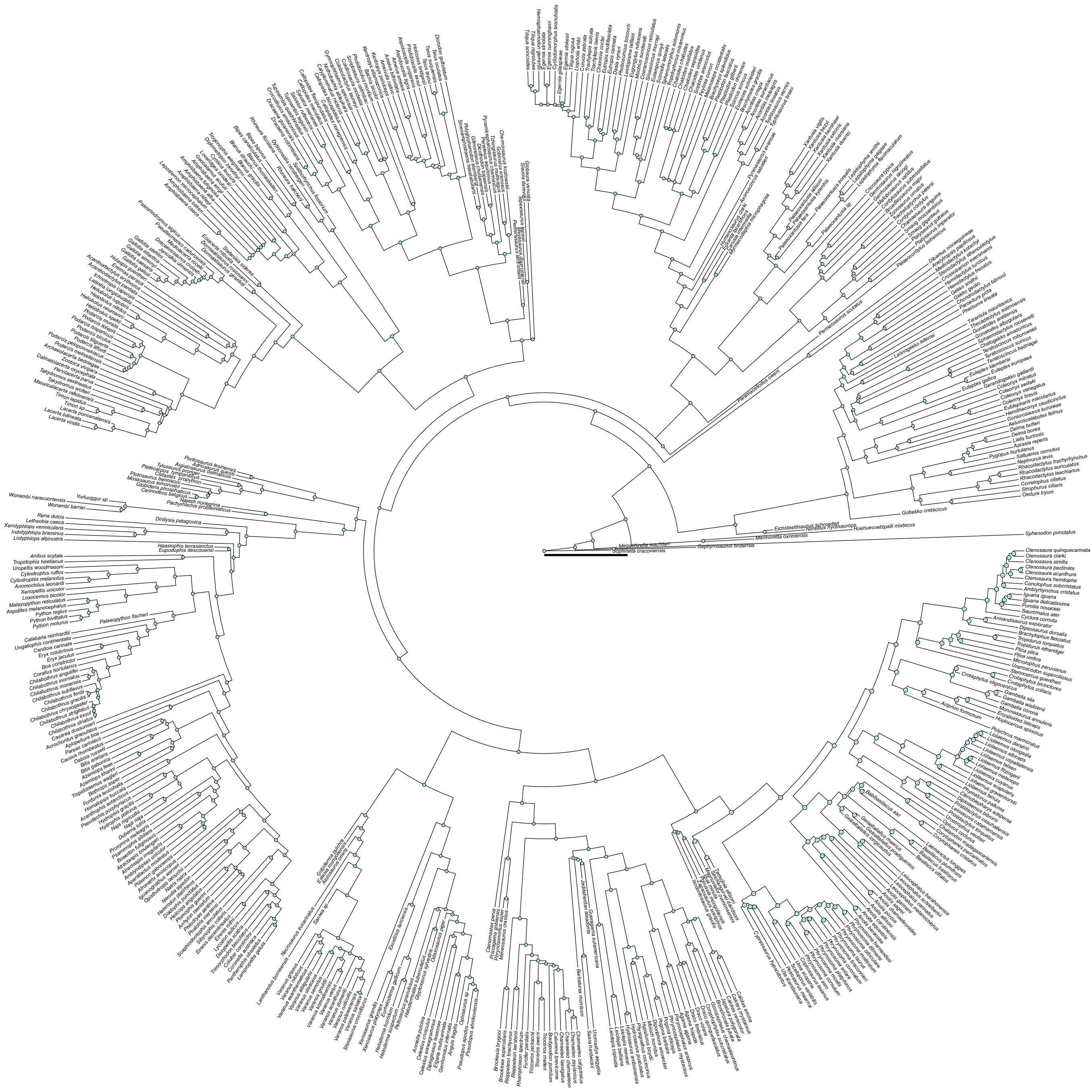

Sphenodon punctatus
Gephyrosaurus bridensis
Marmoretta oxoniensis
Megachirella wachtleri
Sophineta cracoviensis
Eichstaettisaurus schroederi
Norellius nyctisaurops
Huehuecuetzpalli mixtecus
Gobekko cretacicus
Oedura tryoni
Strophurus ciliaris
Correlophus ciliatus
Rhacodactylus leachianus
Rhacodactylus auriculatus
Rhacodactylus trachyrhynchus
Nephrurus levis
Saltuarius cornutus
Pygopus hortulanus
Aprasia repens
Lialis burtonis
Delma borea
Delma butleri
Aeluroscalabotes felinus
Goniurosaurus kuroiwae
Hemitheconyx caudicinctus
Eublepharis macularius
Coleonyx brevis
Coleonyx variegatus
Coleonyx switaki
Coleonyx mitratus
Ctenosaura quinquecarinata
Ctenosaura clarki
Ctenosaura similis
Ctenosaura pectinata
Ctenosaura acanthura
Ctenosaura hemilopha
Conolophus subcristatus
Amblyrhynchus cristatus
Iguana iguana
Iguana delicatissima
Pumilia novaceki
Sauromalus ater
Cyclura cornuta
Armandisaurus explorator
Dipsosaurus dorsalis
Brachylophus fasciatus
Tropidurus torquatus
Tropidurus etheridgei
Plica plica
Plica umbra
Microlophus peruvianus
Uranoscodon superciliosus
Stenocercus guentheri
Crotaphytus oligocenicus
Crotaphytus bicinctores
Crotaphytus collaris
Gambelia sila
Gambelia wislizenii
Gambelia corona
Morunasaurus annularis
Enyalioides laticeps
Hoplocercus spinosus
Aciprion formosum
Wonambi naracoortensis
Yurlunggur sp
Wonambi barriei
Rena dulcis
Letheobia caeca
Xerotyphlops vermicularis
Indotyphlops braminus
Liotyphlops albirostris
Dinilysia patagonica
Haasiophis terrasanctus
Eupodophis descouensi
Anilius scytale
Tropidophis haetianus
Uropeltis woodmasoni
Cylindrophis ruffus
Cylindrophis melanotus
Anomochilus leonardi
Xenopeltis unicolor
Loxocemus bicolor
Malayopython reticulatus
Aspidites melanocephalus
Python regius
Python bivittatus
Python molurus
Palaeopython fischeri
Calabaria reinhardtii
Ungaliophis continentalis
Candoia carinata
Eryx colubrinus
Eryx jaculus
Boa constrictor
Corallus hortulanus

Supplementary Fig. 3 | Time-calibrated dichotomous squamate phylogeny with node nomenclature. Informal super-tree including 545 extant and extinct squamates species and three outgroup species (see Methods). Labels indicate node numbers as referred in the text and Supplementary Information. Scalebar = 50 million years.

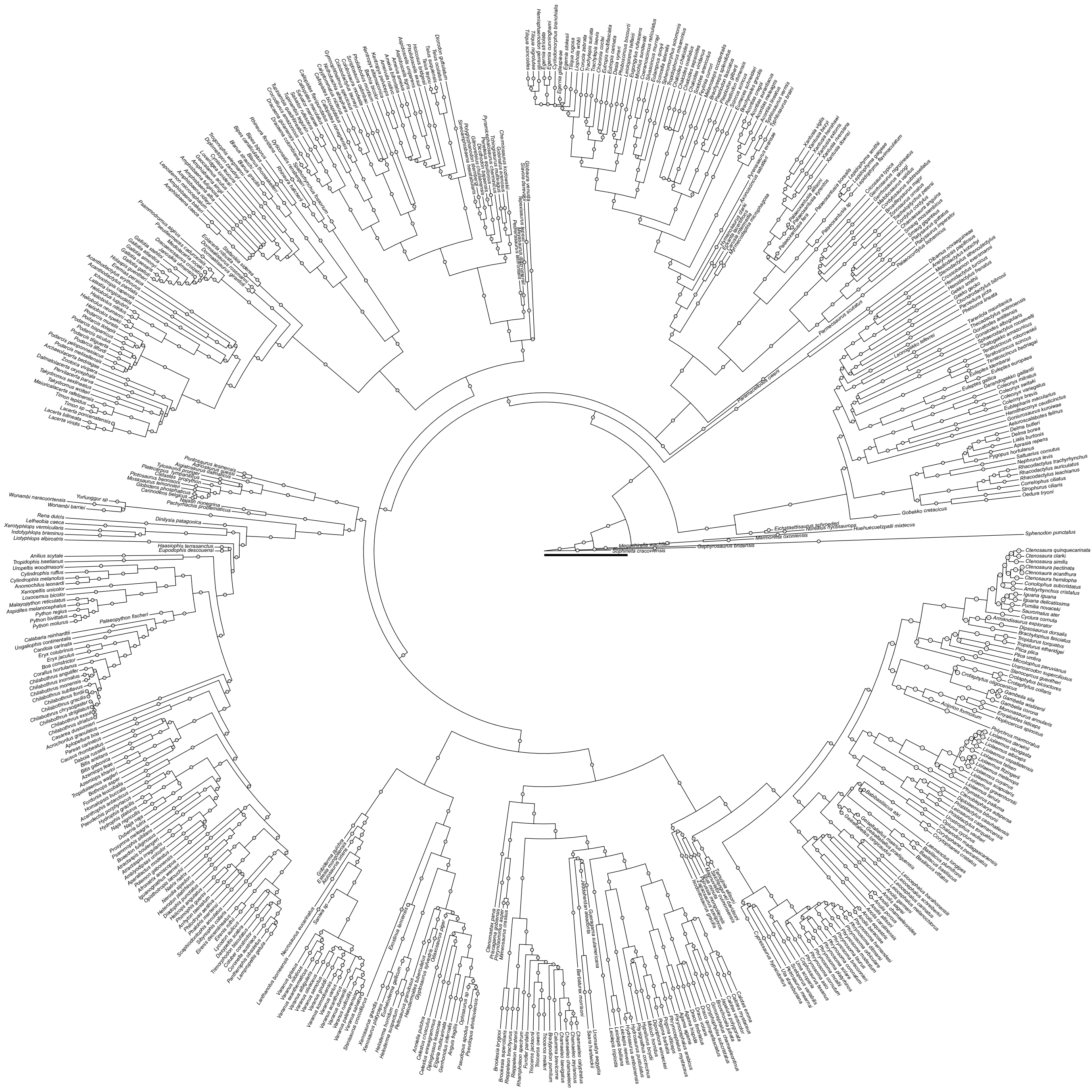

Sphenodon punctatus
Gephyrosaurus bridensis
Marmoretta oxoniensis
Megachirella wachtleri
Sophineta cracoviensis
Eichstaettisaurus schroederi
Norellius nyctisaurops
Huehuecuetzpalli mixtecus
Gobekko cretacicus
Oedura tryoni
Strophurus ciliaris
Correlophus ciliatus
Rhacodactylus leachianus
Rhacodactylus auriculatus
Nephrurus trachyrhynchus
Nephrurus levis
Saltuarius cornutus
Pygopus hortulanus
Aprasia repens
Lialis burtonis
Delma borea
Delma butleri
Aeluroscalabotes felinus
Goniurosaurus kuroiwae
Hemitheconyx caudicinctus
Eublepharis macularius
Coleonyx brevis
Coleonyx variegatus
Coleonyx switaki
Coleonyx mitratus
Ctenosaura quinquecarinata
Ctenosaura clarki
Ctenosaura similis
Ctenosaura pectinata
Ctenosaura acanthura
Ctenosaura hemilopha
Conolophus subcristatus
Amblyrhynchus cristatus
Iguana iguana
Iguana delicatissima
Pumilia novaceki
Sauromalus ater
Cyclura cornuta
Armandisaurus explorator
Dipsosaurus dorsalis
Brachylophus fasciatus
Tropidurus torquatus
Tropidurus etheridgei
Plica plica
Plica umbra
Microlophus peruvianus
Uranoscodon superciliosus
Stenocercus guentheri
Crotaphytus bicinctores
Crotaphytus collaris
Gambelia sila
Gambelia wislizenii
Gambelia corona
Morunasaurus annularis
Enyalioides laticeps
Hoplocercus spinosus
Aciprion formosum
Wonambi naracoortensis
Yurlunggur sp
Wonambi barriei
Rena dulcis
Letheobia caeca
Xerotyphlops vermicularis
Indotyphlops braminus
Liotyphlops albirostris
Dinilysia patagonica
Haasiophis terrasanctus
Eupodophis descouensi
Anilius scytale
Tropidophis haetianus
Uropeltis woodmasoni
Cylindrophis ruffus
Cylindrophis melanotus
Anomochilus leonardi
Xenopeltis unicolor
Loxocemus bicolor
Malayopython reticulatus
Aspidites melanocephalus
Python regius
Python bivittatus
Python molurus
Palaeopython fischeri
Calabaria reinhardtii
Ungaliophis continentalis
Candoia carinata
Eryx colubrinus
Eryx jaculus
Boa constrictor
Corallus hortulanus

Supplementary Fig. 4 | Time-calibrated dichotomous squamate phylogeny with branch nomenclature. Informal super-tree including 545 extant and extinct squamates species and three outgroup species (see Methods). Labels indicate branch numbers as referred in the text and Supplementary Information. Scalebar = 50 million years.

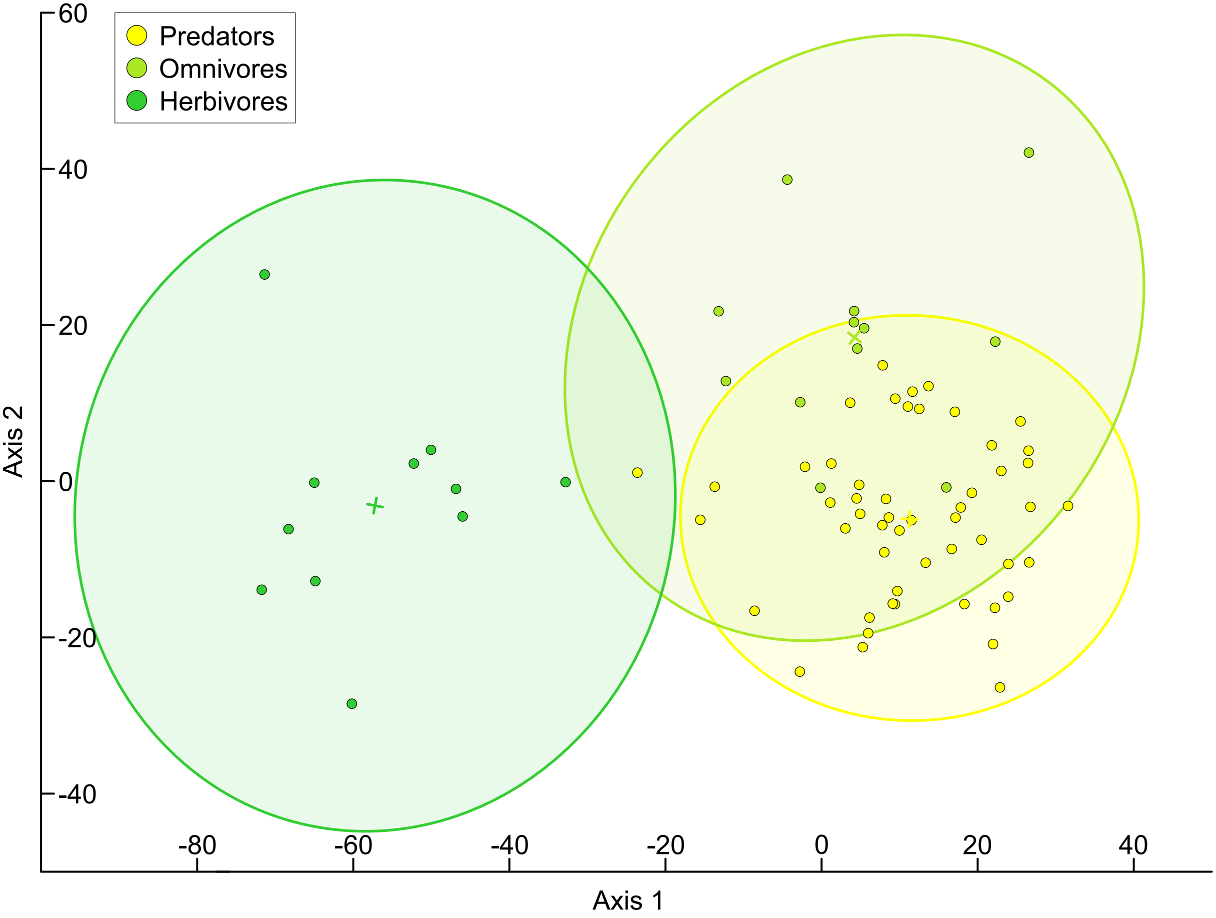
Predators
Omnivores
Herbivores
Axis 2
60
40
20
0
-20
-40
-80
-60
-40
-20
0
20
40
Axis 1

Supplementary Fig. 5 | Discriminant analysis of dietary categories in the 2D tooth morphospace. Discriminant Function Analysis (DFA) based on the fitted penalized likelihood phylogenetic multivariate linear model of PC scores for the multicuspid teeth of 75 extant and fossil squamate species grouped by diet (predators, n = 52 (including insectivores, n = 50, and carnivores, n = 2); omnivores, n = 12; herbivores, n = 11) with 95% confidence ellipses (crosses indicate centroids). Axis 1 = 89.68% of between-group variance, Axis 2 = 10.32%. Source data are provided as a Source Data file.

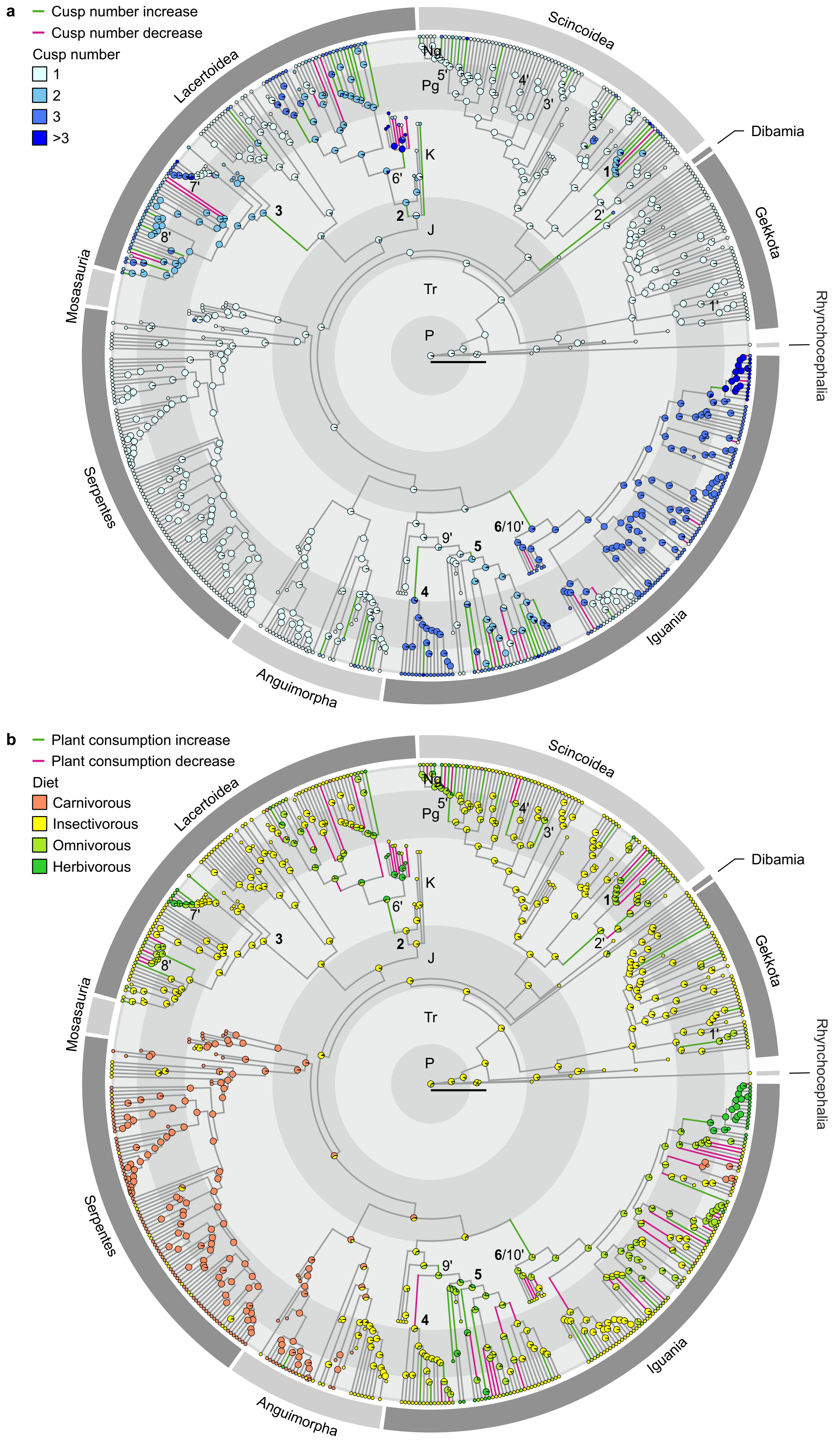
a
Cusp number increase
Cusp number decrease
Cusp number
1
2
3
>3
Scincoidea
Dibamia
Gekkota
Rhynchocephalia
Iguania
Anguimorpha
Serpentes
Mosasauria
Lacertoidea
Ng
Pg
K
J
Tr
P
1
2
3
4
5
6/10'
1'
2'
3'
4'
5'
6'
7'
8'
9'
b
Plant consumption increase
Plant consumption decrease
Diet
Carnivorous
Insectivorous
Omnivorous
Herbivorous
Scincoidea
Dibamia
Gekkota
Rhynchocephalia
Iguania
Anguimorpha
Serpentes
Mosasauria
Lacertoidea

Supplementary Fig. 6 | Squamate dental and dietary evolution. Known and Maximum Likelihood ancestral state reconstructions of tooth complexity (**a**) and diet (**b**) in squamates. Pie charts indicate the relative likelihood of each character state at the corresponding node. Branch tip circles indicate character state at tips. Coloured branches indicate an increase or a decrease in tooth complexity/plant consumption. 1: Gerrhosauridae (node 616; see Supplementary Figure 1). 2: Teiioidea + Polyglyphanodontia (informally Teiioidea *sensu lato*; node 686). 3: total group Lacertidae (informally Lacertidae *sensu lato*; node 740). 4: Chamaeleonidae (node 930). 5: non-Uromastycinae agamids (informally Agamidae *sensu stricto*; node 949). 6: total group Pleurodonta (node 971). 1': unnamed clade including the most recent common ancestor (MRCA) to *Correlophus ciliatus* and *Rhacodactylus auriculatus* and all its descendants (node 563). 2': Cordyloidea (node 609). 3': unnamed clade including the MRCA to *Eumeces schneideri* and *Scincus scincus* and all its descendants (node 652). 4': *Chalcides* (node 657). 5': Egerniinae (node 674). 6': unnamed clade including the MRCA to *Polyglyphanodon sternbergi* and *Teius teyou* and all its descendants (node 688). 7': *Gallotia* (node 750). 8': *Podarcis* (node 765). 9': crown Acrodonta (node 929). 10': total group Pleurodonta (node 971). P: Permian. Tr: Triassic. J: Jurassic. K: Cretaceous. Pg: Paleogene. Ng: Neogene. Scalebar = 50 million years.

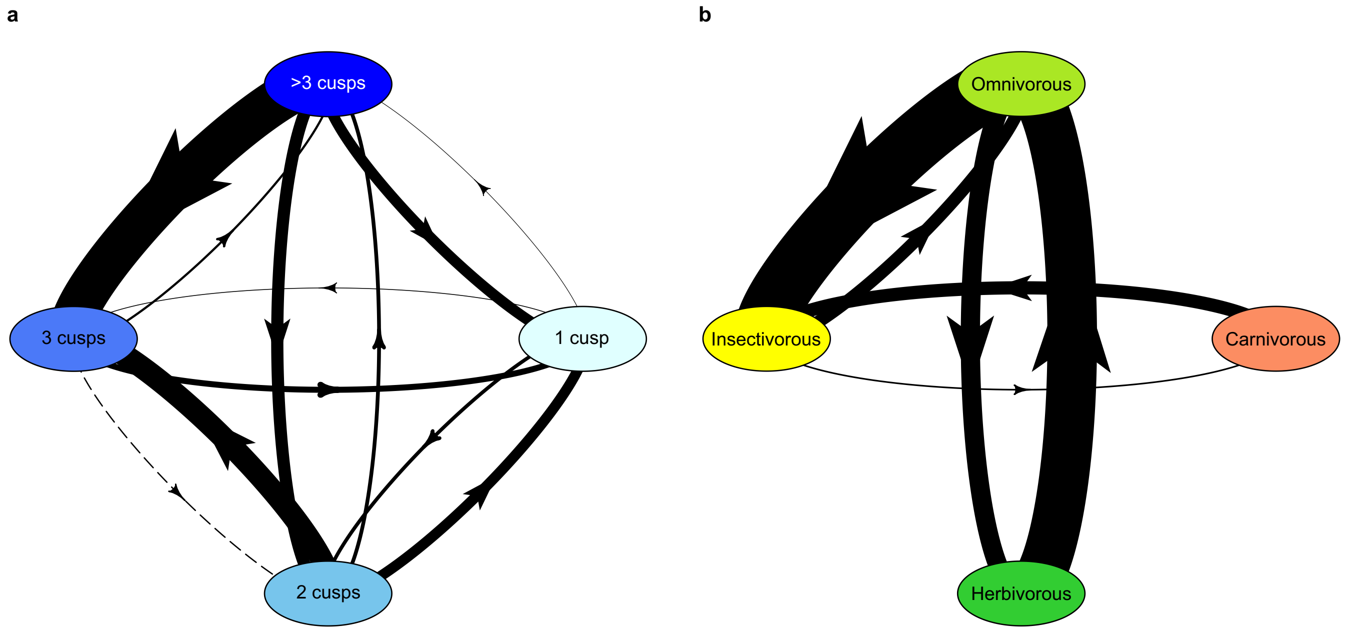

a
b
>3 cusps
3 cusps
1 cusp
2 cusps
Omnivorous
Insectivorous
Carnivorous
Herbivorous

Supplementary Fig. 7 | Transition models used for ancestral state reconstructions. Relative character transition rates for tooth complexity (**a**) and diet (**b**). Arrow widths are scaled by the log-transformed rates of transition. The orientation of arrows denotes the direction of character transitions. Note: the transition from three-cusped to two-cusped teeth is represented with a dotted line, due to its relative rate being negligible compared to all other transition rates.

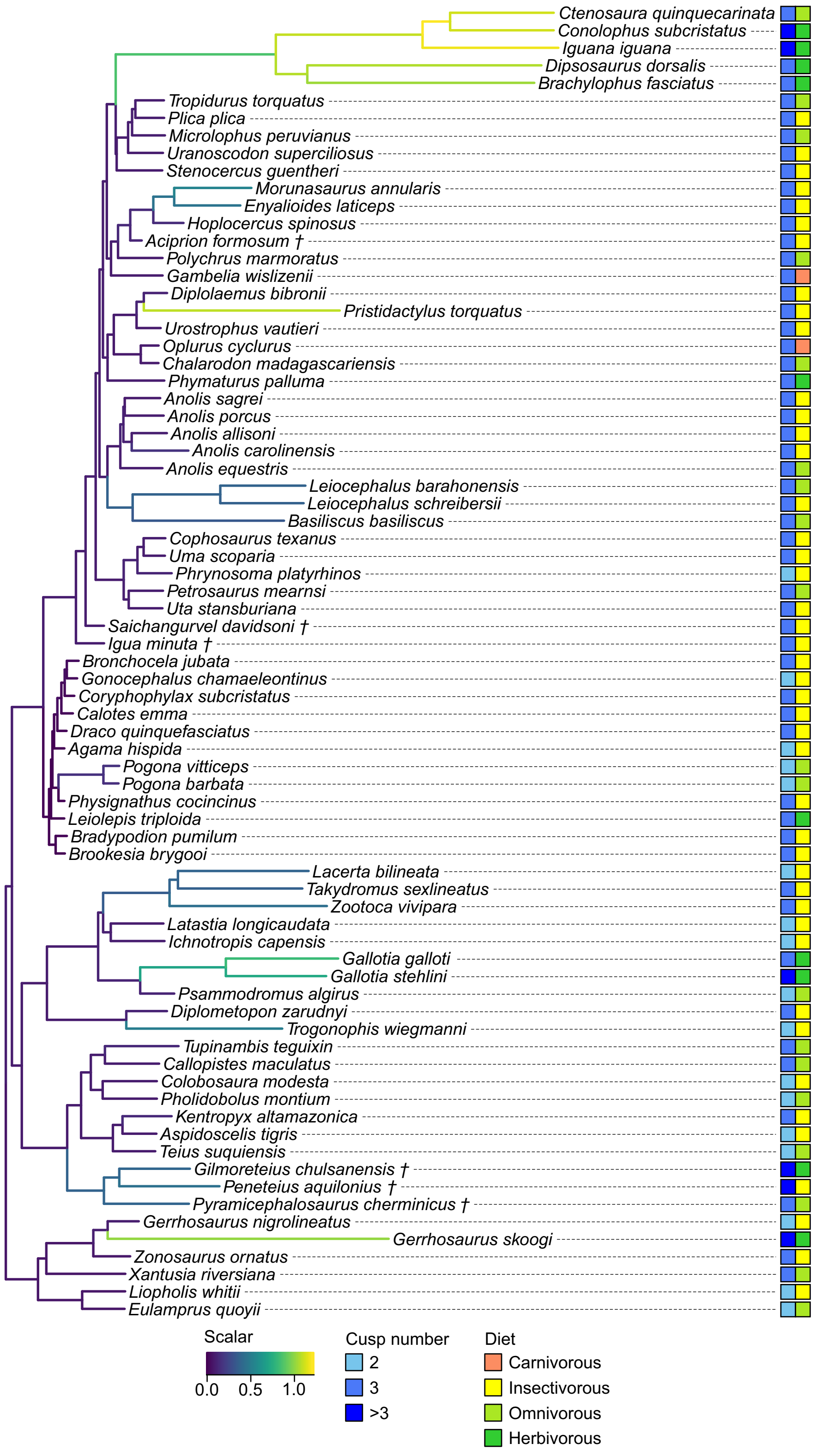

Ctenosaura quinquecarinata
Conolophus subcristatus
Iguana iguana
Dipsosaurus dorsalis
Brachylophus fasciatus
Tropidurus torquatus
Plica plica
Microlophus peruvianus
Uranoscodon superciliosus
Stenocercus guentheri
Morunasaurus annularis
Enyalioides laticeps
Hoplocercus spinosus
Aciprion formosum †
Polychrus marmoratus
Gambelia wislizenii
Diplolaemus bibronii
Pristidactylus torquatus
Urostrophus vautieri
Oplurus cyclurus
Chalarodon madagascariensis
Phymaturus palluma
Anolis sagrei
Anolis porcus
Anolis allisoni
Anolis carolinensis
Anolis equestris
Leiocephalus barahonensis
Leiocephalus schreibersii
Basiliscus basiliscus
Cophosaurus texanus
Uma scoparia
Phrynosoma platyrhinos
Petrosaurus mearnsi
Uta stansburiana
Saichangurvel davidsoni †
Igua minuta †
Bronchocela jubata
Gonocephalus chamaeleontinus
Coryphophylax subcristatus
Calotes emma
Draco quinquefasciatus
Agama hispida
Pogona vitticeps
Pogona barbata
Physignathus cocincinus
Leiolepis triploida
Bradypodion pumilum
Brookesia brygooi
Lacerta bilineata
Takydromus sexlineatus
Zootoca vivipara
Latastia longicaudata
Ichnotropis capensis
Gallotia galloti
Gallotia stehlini
Psammodromus algirus
Diplometopon zarudnyi
Trogonophis wiegmanni
Tupinambis teguixin
Callopistes maculatus
Colobosaura modesta
Pholidobolus montium
Kentropyx altamazonica
Aspidoscelis tigris
Teius suquiensis
Gilmoreteius chulsanensis †
Peneteius aquilonius †
Pyramicephalosaurus cherminicus †
Gerrhosaurus nigrolineatus
Gerrhosaurus skoogi
Zonosaurus ornatus
Xantusia riversiana
Liopholis whitii
Eulamprus quoyii
Scalar
0.0 0.5 1.0
Cusp number
2
3
>3
Diet
Carnivorous
Insectivorous
Omnivorous
Herbivorous

Supplementary Fig. 8 | Rates of 2D tooth shape evolution among 75 squamate species with multicuspid teeth. Branch lengths are transformed by the mean of the respective posterior distribution of scalars generated under a variable rates model, reflecting changes in the rate of shape evolution. Dagger = extinct taxon. Marginal log-likelihood (logLik) of the "variable rates" model logLik = -368.91; null constant rate model (Brownian Motion): logLik = -528.41; log Bayes Factor = 319.

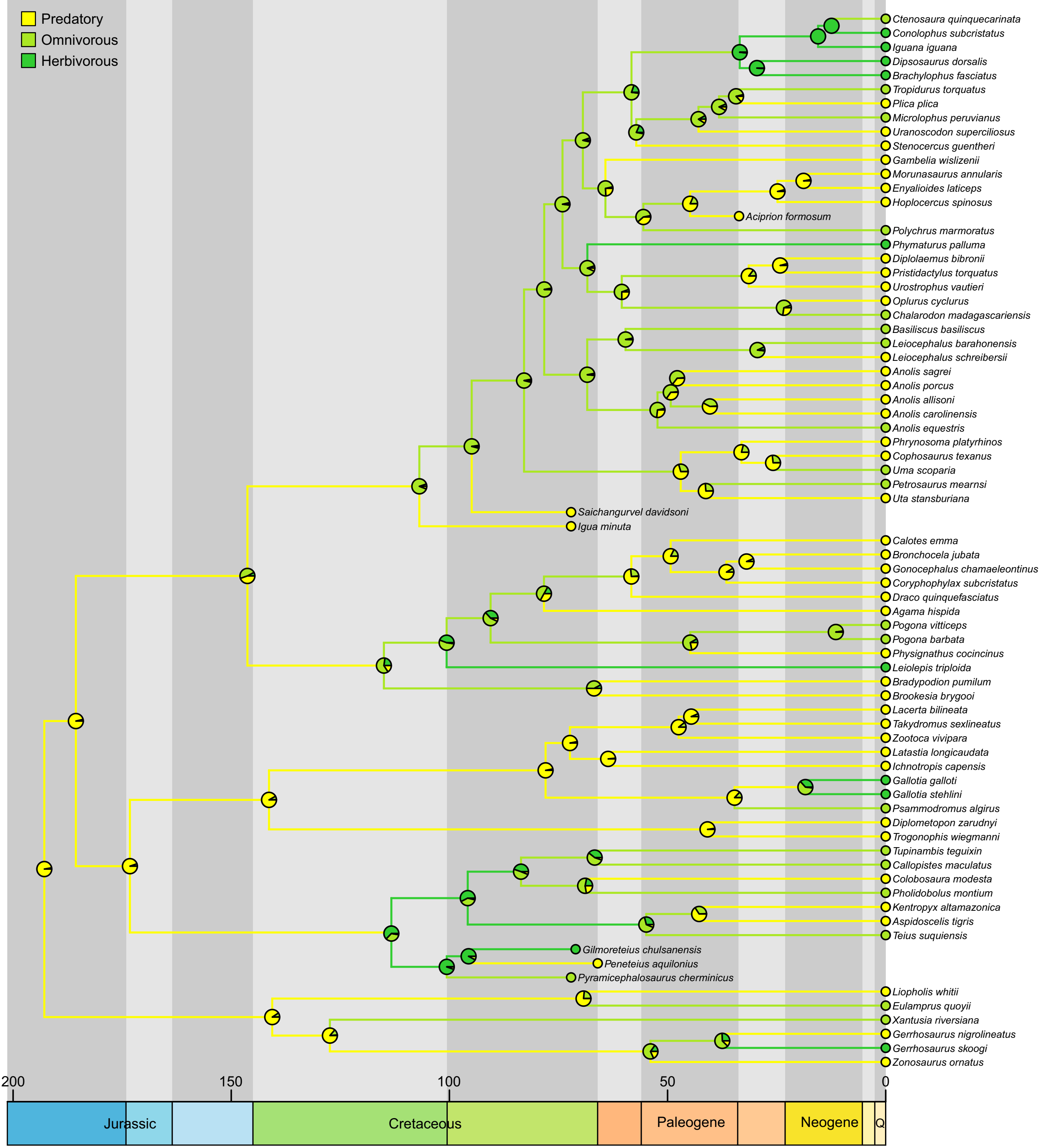

Predatory
Omnivorous
Herbivorous
Ctenosaura quinquecarinata
Conolophus subcristatus
Iguana iguana
Dipsosaurus dorsalis
Brachylophus fasciatus
Tropidurus torquatus
Plica plica
Microlophus peruvianus
Uranoscodon superciliosus
Stenocercus guentheri
Gambelia wislizenii
Morunasaurus annularis
Enyalioides laticeps
Hoplocercus spinosus
Aciprion formosum
Polychrus marmoratus
Phymaturus palluma
Diplolaemus bibronii
Pristidactylus torquatus
Urostrophus vautieri
Oplurus cyclurus
Chalarodon madagascariensis
Basiliscus basiliscus
Leiocephalus barahonensis
Leiocephalus schreibersii
Anolis sagrei
Anolis porcus
Anolis allisoni
Anolis carolinensis
Anolis equestris
Phrynosoma platyrhinos
Cophosaurus texanus
Uma scoparia
Petrosaurus mearnsi
Uta stansburiana
Saichangurvel davidsoni
Igua minuta
Calotes emma
Bronchocela jubata
Gonocephalus chamaeleontinus
Coryphophylax subcristatus
Draco quinquefasciatus
Agama hispida
Pogona vitticeps
Pogona barbata
Physignathus cocincinus
Leiolepis triploida
Bradypodion pumilum
Brookesia brygooi
Lacerta bilineata
Takydromus sexlineatus
Zootoca vivipara
Latastia longicaudata
Ichnotropis capensis
Gallotia galloti
Gallotia stehlini
Psammodromus algirus
Diplometopon zarudnyi
Trogonophis wiegmanni
Tupinambis teguixin
Callopistes maculatus
Colobosaura modesta
Pholidobolus montium
Kentropyx altamazonica
Aspidoscelis tigris
Teius suquiensis
Gilmoreteius chulsanensis
Peneteius aquilonius
Pyramicephalosaurus cherminicus
Liopholis whitii
Eulamprus quoyii
Xantusia riversiana
Gerrhosaurus nigrolineatus
Gerrhosaurus skoogi
Zonosaurus ornatus
200
150
100
50
0
Jurassic
Cretaceous
Paleogene
Neogene
Q

Supplementary Fig. 9 | Dietary evolution among the geometric morphometrics taxonomic subset. Known and Maximum Likelihood ancestral state reconstructions of diet (predatory, omnivorous, herbivorous) for 75 squamate species bearing multiple-cusped teeth. Pie charts indicate the relative likelihood of each character state at the corresponding node. Branch tip circles indicate character state at tips. Branch colours indicate the discrete stochastic mapping used in continuous multivariate models of trait evolution based on tip and node state (see Supplementary Tables 10 and 11).

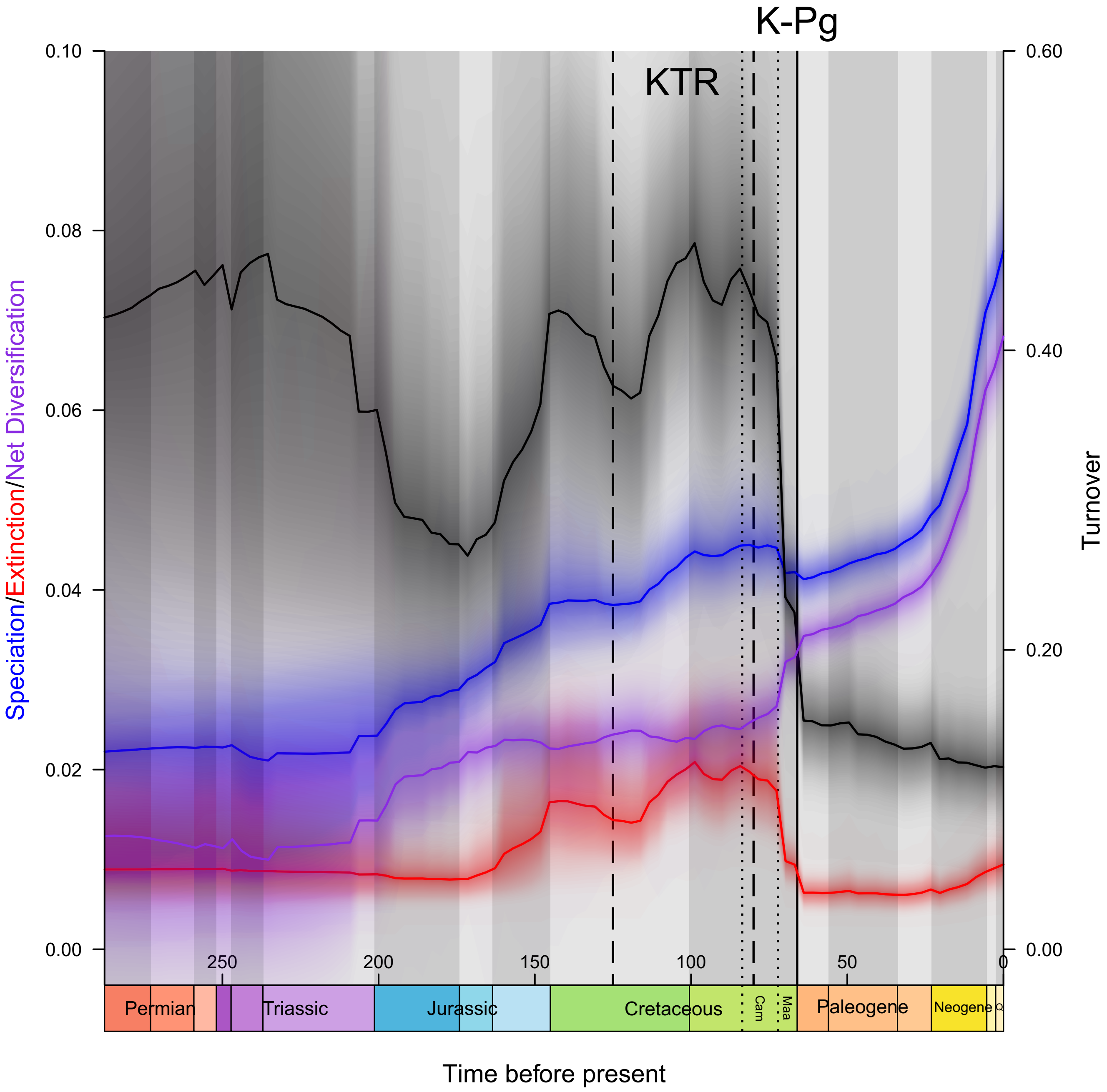

K-Pg
KTR
Speciation/Extinction/Net Diversification
Turnover
0.10
0.08
0.06
0.04
0.02
0.00
0.60
0.40
0.20
0.00
250
200
150
100
50
0
Permian
Triassic
Jurassic
Cretaceous
Cam
Maa
Paleogene
Neogene
Q
Time before present

Supplementary Fig. 10 | Patterns of squamate diversification. Mean rates of squamate speciation, extinction, and net diversification (in My^{-1}), and turnover (extinction/speciation) through time. Speciation locally peaked at the end of the KTR, in the Campanian. Extinction and turnover reached their all-time maximum during the KTR, at the beginning of the Late Cretaceous (Cenomanian). Speciation, extinction, and turnover all drop in the Maastrichtian, prior to and across the Cretaceous-Paleogene mass extinction. Shaded areas: 95% confidence interval. KTR: Cretaceous Terrestrial Revolution (125–80 Ma). K-Pg: Cretaceous–Paleogene boundary. Cam: Campanian. Maa: Maastrichtian. Q: Quaternary.

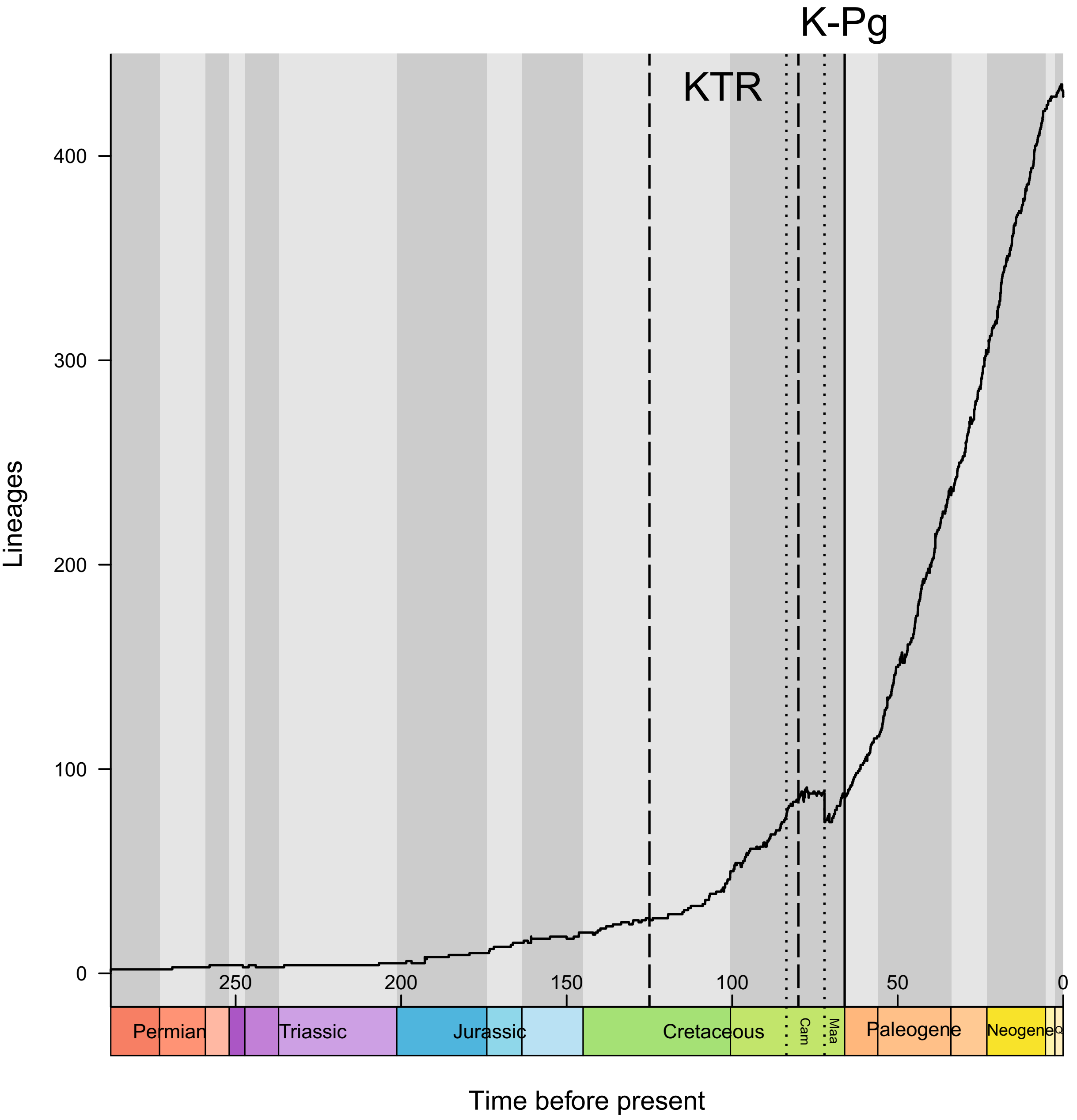
K-Pg
KTR
Lineages
400
300
200
100
0
250
200
150
100
50
0
Permian
Triassic
Jurassic
Cretaceous
Cam
Maa
Paleogene
Neogene
Q
Time before present

Supplementary Fig. 11 | Squamate diversity through time. Absolute number of lineages through time based on the time-calibrated dichotomous squamate super-tree. Squamate diversity locally peaked immediately following the end of the KTR, in the Campanian. KTR: Cretaceous Terrestrial Revolution (125–80 Ma). K-Pg: Cretaceous–Paleogene boundary. Cam: Campanian. Maa: Maastrichtian. Q: Quaternary.

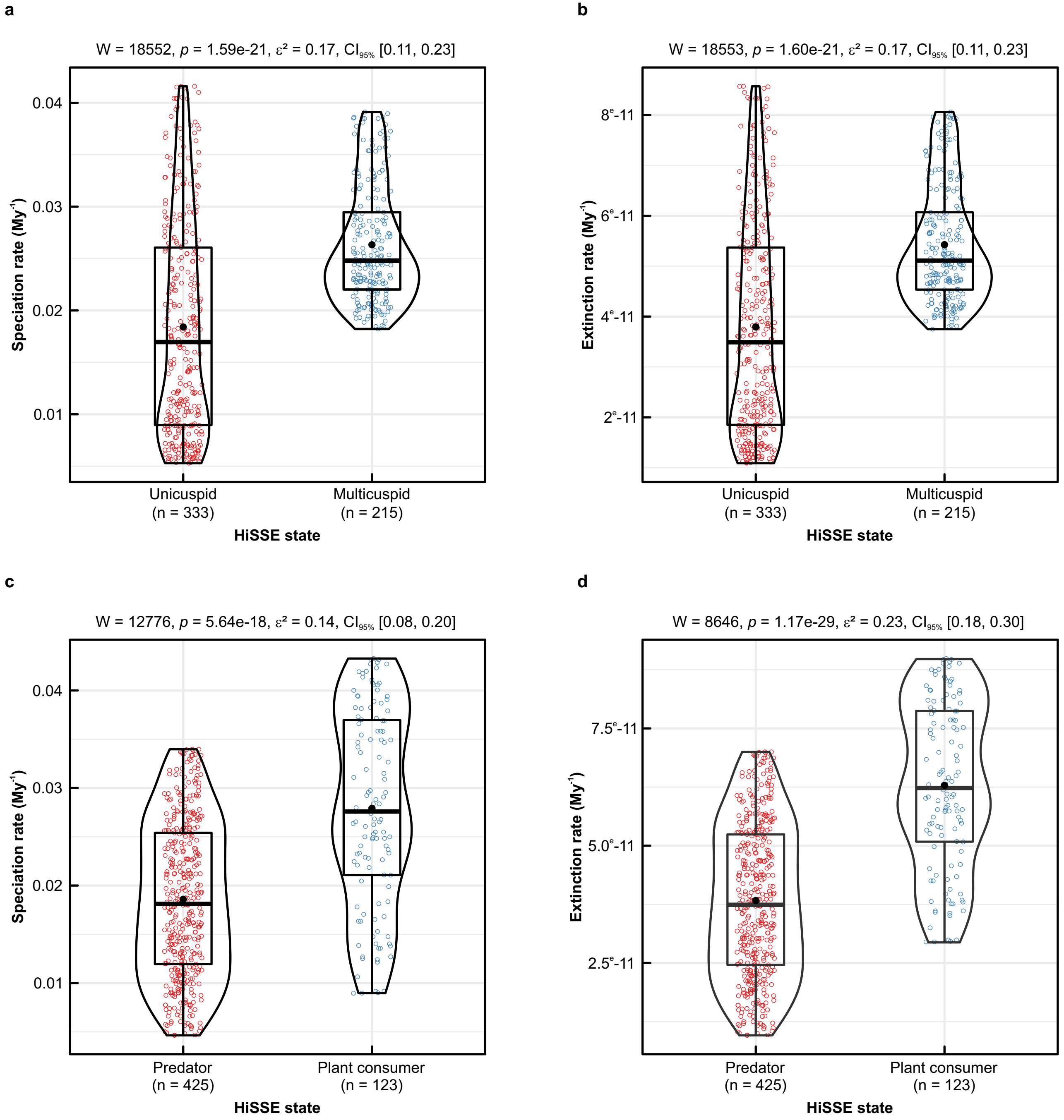

a
W = 18552, p = 1.59e-21, ε² = 0.17, CI95% [0.11, 0.23]
Speciation rate (My-1)
0.04
0.03
0.02
0.01
Unicuspid (n = 333)
Multicuspid (n = 215)
HiSSE state
b
W = 18553, p = 1.60e-21, ε² = 0.17, CI95% [0.11, 0.23]
Extinction rate (My-1)
8e-11
6e-11
4e-11
2e-11
Unicuspid (n = 333)
Multicuspid (n = 215)
HiSSE state
c
W = 12776, p = 5.64e-18, ε² = 0.14, CI95% [0.08, 0.20]
Speciation rate (My-1)
0.04
0.03
0.02
0.01
Predator (n = 425)
Plant consumer (n = 123)
HiSSE state
d
W = 8646, p = 1.17e-29, ε² = 0.23, CI95% [0.18, 0.30]
Extinction rate (My-1)
7.5e-11
5.0e-11
2.5e-11
Predator (n = 425)
Plant consumer (n = 123)
HiSSE state

Supplementary Fig. 12 | Squamate diversification rates among different levels of tooth complexity and diet Speciation (**a–c**) and extinction rates (**b–d**) per tooth complexity or diet character state for the best-supported model of trait-dependent diversification. Violin plots indicate the density of data points. Boxes include 50% of the data points (25th to 75th percentile), with the black line and dot indicating the median and mean, respectively. Whiskers incorporate the whole range of the data (minimum to maximum value; no outliers detected). All pairs are statistically significantly different (two-sided Wilcoxon–Mann–Whitney test); see panels for their respective W statistic and effect size (ε^2), including 2.5^{th} and 97.5^{th} confidence interval percentiles ($CI_{95\%}$) in brackets.

Supplementary Tables

Supplementary Table 1 | Tooth complexity of squamates analysed by dietary category

Group 1	Group 2	Sample size	W	*p*-value
Carnivores	Herbivores	154	490	**4.9e-21**
Carnivores	Insectivores	425	12458	**1.8e-08**
Carnivores	Omnivores	197	1583	**7.3e-21**
Herbivores	Insectivores	351	10188	**8.2e-13**
Herbivores	Omnivores	123	2330	**4.6e-04**
Insectivores	Omnivores	394	7543	**4.5e-10**

Two-sided pairwise Wilcoxon–Mann–Whitney tests on the tooth complexity level of 548 species grouped by diet (carnivores, n = 114; insectivores, n = 311; omnivores, n = 83; herbivores, n= 40), with W statistic and Bonferroni-corrected p values. Significant differences ($p < 0.05$) are in bold.

Supplementary Table 2 | Pairwise comparisons of dietary categories in 2D tooth morphospace

Group 1	Group 2	Sample size	V	*p*-value
Herbivores	Omnivores	23	0.64	**4.0e-04**
Herbivores	Predators	63	0.76	**1.0e-04**
Omnivores	Predators	64	0.29	0.82
Herbivores + Omnivores	Predators	75	0.65	**1.0e-04**
Herbivores	Omnivores + Predators	75	0.73	**1.0e-04**

Multivariate general linear hypothesis testing through phylogenetic penalized likelihood MANOVA of PC scores for the multicuspid teeth of 75 extant and fossil squamate species grouped by diet (predators, n = 52 (including insectivores, n = 50, and carnivores, n = 2); omnivores, n = 12; herbivores, n = 11). We assessed statistical significance with a one-sided Pillai trace test over 10,000 permutations of the Pillai trace (V). Significant differences ($p < 0.05$) are in bold; p values are uncorrected.

Supplementary Table 3 | Model selection for ancestral character states reconstructions (four-state diet).

Model	Number of transitions	Number of rates	AICc (tooth complexity)	ωAICc (tooth complexity)	AICc (diet)	ωAICc (diet)
**ARD**	**12**	**12**	**661.584**	**~1**	815.412	~0.375
SYM	12	6	710.564	< 0.001	877.410	< 0.001
ER	12	1	737.734	< 0.001	928.694	< 0.001
**progARD**	**6**	**6**	NA	NA	**814.387**	**~0.625**
progSYM	6	3	NA	NA	877.601	< 0.001
progER	6	1	NA	NA	1018.540	< 0.001

Description of model parameters and relative goodness of fit. Corrected Akaike Information Criterion (AICc) values and AICc model weights (ωAICc) for the tooth complexity and diet dataset. The model with the highest AICc weight is highlighted in bold. ARD: all rates different. SYM: symmetric rates (forces rate[A→B] = rate[B→A]). ER: equal rates. "prog" models correspond to the three-rate variants of the dietary evolution model tested in this study.

Supplementary Table 4 | Transition models used for the stochastic character mapping of cusp number and four-state diet.

Character	q12	q13	q14	q21	q23	q24	q31	q32	q34	q41	q42	q43
Cusp number	1.7e-03	4.5e-04	1.3e-04	5.2e-03	1.1e-02	1.9e-03	3.0e-03	7.6e-14	1.1e-03	5.4e-03	6.1e-03	2.2e-02
Diet	6.6e-03	0	0	8.5e-04	6.3e-03	0	0	3.0e-02	9.6e-03	0	0	2.5e-02

q: relative transition rate between character states. Character states are encoded as follows: Cusp number (1 – single-cusped, 2 – two-cusped, 3 – three-cusped, 4 – more than three cusps); Diet (1 – carnivore, 2 – insectivore, 3 – omnivore, 4 – herbivore).

Supplementary Table 5 | Events of paired increases or decreases in tooth complexity and plant consumption. Transition locations are referred to by their branch number (see Supplementary Figure 2).

Tooth complexity transition	Branch	Diet transition	Branch	Transition type	Pair type
1 cusp to 2 cusps	132	insectivore to omnivore	118	increase	plant consumption first
2 cusps to 1 cusp	139	omnivore to insectivore	139	decrease	co-occurrent
2 cusps to >3 cusps	143	omnivore to herbivore	143	increase	co-occurrent
1 cusp to 2 cusps	154	insectivore to herbivore	154	increase	co-occurrent
1 cusp to 3 cusps	167	insectivore to omnivore	167	increase	co-occurrent
1 cusp to 2 cusps	230	insectivore to omnivore	230	increase	co-occurrent
1 cusp to >3 cusps	250	insectivore to herbivore	250	increase	co-occurrent
1 cusp to 3 cusps	263	omnivore to herbivore	263	increase	co-occurrent
1 cusp to 2 cusps	272	insectivore to omnivore	254	increase	plant consumption first
1 cusp to 2 cusps	280	insectivore to herbivore	284	increase	tooth complexity first
2 cusps to >3 cusps	285	insectivore to herbivore	284	increase	plant consumption first
>3 cusps to 3 cusps	286	herbivore to omnivore	286	decrease	co-occurrent
>3 cusps to 1 cusp	289	herbivore to insectivore	289	decrease	co-occurrent
>3 cusps to 1 cusp	292	herbivore to insectivore	292	decrease	co-occurrent
>3 cusps to 2 cusps	293	herbivore to omnivore	293	decrease	co-occurrent
2 cusps to 3 cusps	299	omnivore to herbivore	299	increase	co-occurrent
2 cusps to 1 cusp	326	omnivore to insectivore	326	decrease	co-occurrent
2 cusps to 1 cusp	330	omnivore to insectivore	328	decrease	plant consumption first
2 cusps to 1 cusp	334	omnivore to insectivore	328	decrease	plant consumption first
2 cusps to 3 cusps	347	omnivore to herbivore	354	increase	tooth complexity first
3 cusps to 1 cusp	357	omnivore to insectivore	355	decrease	plant consumption first
1 cusp to 2 cusps	398	insectivore to omnivore	408	increase	tooth complexity first
1 cusp to 2 cusps	398	insectivore to omnivore	447	increase	tooth complexity first
1 cusp to >3 cusps	417	insectivore to omnivore	417	increase	co-occurrent
1 cusp to >3 cusps	417	omnivore to herbivore	418	increase	tooth complexity first
>3 cusps to 3 cusps	419	herbivore to omnivore	424	decrease	tooth complexity first
2 cusps to 3 cusps	463	insectivore to omnivore	464	increase	tooth complexity first
2 cusps to 3 cusps	475	insectivore to omnivore	478	increase	tooth complexity first
2 cusps to 3 cusps	484	insectivore to omnivore	484	increase	co-occurrent
1 cusp to 3 cusps	783	insectivore to omnivore	794	increase	tooth complexity first
1 cusp to 3 cusps	783	insectivore to omnivore	800	increase	tooth complexity first
1 cusp to 3 cusps	783	insectivore to omnivore	805	increase	tooth complexity first
1 cusp to 2 cusps	822	omnivore to herbivore	823	increase	tooth complexity first
1 cusp to 2 cusps	822	insectivore to omnivore	782	increase	plant consumption first
2 cusps to 3 cusps	824	omnivore to herbivore	823	increase	plant consumption first
2 cusps to 3 cusps	825	omnivore to herbivore	823	increase	plant consumption first
3 cusps to 1 cusp	826	herbivore to omnivore	825	decrease	plant consumption first
2 cusps to 3 cusps	832	omnivore to herbivore	830	increase	plant consumption first
2 cusps to 1 cusp	837	omnivore to insectivore	837	decrease	co-occurrent
2 cusps to 1 cusp	846	omnivore to insectivore	845	decrease	plant consumption first
2 cusps to 1 cusp	854	omnivore to insectivore	852	decrease	plant consumption first
2 cusps to 3 cusps	864	insectivore to omnivore	865	increase	tooth complexity first
1 cusp to 3 cusps	869	insectivore to omnivore	869	increase	co-occurrent
1 cusp to 3 cusps	869	omnivore to herbivore	1037	increase	tooth complexity first
3 cusps to 1 cusp	874	omnivore to insectivore	874	decrease	co-occurrent
3 cusps to 1 cusp	890	omnivore to insectivore	883	decrease	plant consumption first
3 cusps to 1 cusp	895	omnivore to insectivore	883	decrease	plant consumption first
3 cusps to 1 cusp	959	omnivore to insectivore	954	decrease	plant consumption first
3 cusps to 1 cusp	972	omnivore to insectivore	967	decrease	plant consumption first
3 cusps to 1 cusp	1021	omnivore to carnivore	1012	decrease	plant consumption first
3 cusps to >3 cusps	1043	omnivore to herbivore	1037	increase	plant consumption first
>3 cusps to 3 cusps	1067	herbivore to omnivore	1067	decrease	co-occurrent

Supplementary Table 6 | Tests of correlated and uncorrelated models of evolution of tooth complexity and diet in Squamata.

Tooth complexity binarization	Diet binarization	Independent model logLik	Dependent model logLik	logBF
One cusp vs two cusps or more	Omnivores and herbivores vs carnivores and insectivores	-446.16	-435.74	**20.85**
One cusp vs two cusps or more	Herbivores vs other diets	-317.08	-317.97	-1.78
One or two cusps vs three or more	Omnivores and herbivores vs carnivores and insectivores	-432.12	-417.81	**28.62**
One or two cusps vs three or more	Herbivores vs other diets	-303.18	-301.51	3.32
Three cusps or less vs four or more	Omnivores and herbivores vs carnivores and insectivores	-303.15	-304.83	-3.37
Three cusps or less vs four or more	Herbivores vs other diets	-174.33	-162.91	**22.84**

Marginal log-likelihoods (logLik) for independent and dependent models of character evolution, with their associated log Bayes Factor (logBF), over three binarizations of tooth complexity, two binarizations of diet, and their combinations. Values in bold (logBF > 10) indicate very strong statistical support for the more complex model (*i.e.*, the dependent model).

Supplementary Table 7 | Test of heterogeneity in relative character transition rates of tooth complexity and diet.

Character binarization	Constant model logLik	Variable rates model logLik	logBF
One cusp vs two cusps or more	-203.86	-198.08	**11.56**
One or two cusps vs three or more	-189.93	-178.27	**23.31**
Three cusps or less vs four or more	-61.29	-62.36	-2.14
Omnivores and herbivores vs other diets	-241.64	-232.49	**18.30**
Herbivores vs other diets	-112.72	-108.53	8.37

Marginal log-likelihoods (logLik) for constant and "variable rates" models of character evolution (allowing local heterogeneity in the character transition rates), with their associated log Bayes Factor (logBF), over three binarizations of tooth complexity and two of diet. Values in bold (logBF > 10) indicate very strong statistical support for the more complex model (*i.e.*, the "variable rates" model).

Supplementary Table 8 | Model selection for three-state diet ancestral character states reconstructions.

Model	Number of transitions	Number of rates	AICc	ωAICc
ARD	6	6	141.805	0.087
SYM	6	3	141.729	0.090
ER	6	1	145.352	0.015
**progARD**	**4**	**4**	**137.345**	**0.808**
progSYM	4	2	168.959	< 0.001
progER	4	1	166.847	< 0.001

Description of model parameters and relative goodness of fit. Corrected Akaike Information Criterion (AICc) values and AICc model weights (ωAICc) for the tooth complexity and diet dataset. The model with the highest AICc weight is highlighted in bold. ARD: all rates different. SYM: symmetric rates (forces rate[A→B] = rate[B→A]). ER: equal rates. "prog" models correspond to the three-rate variants of the dietary evolution model tested in this study adapted to a three-state diet.

Supplementary Table 9 | Transition model used for stochastic character mapping of three-state diet.

Character	q12	q13	q21	q23	q31	q32
Diet	3.6^{e}-03	0	2.6^{e}-02	9.0^{e}-03	0	2.6^{e}-02

q: relative transition rate between character states. Character states are encoded as follows: 1 – predatory, 2 – omnivore, 3 – herbivore.

Supplementary Table 10 | Model selection for multivariate continuous trait evolution

Model	Rank	Multiple optima	df	AICc	ωAICc
BM1	6	No	20	873.224	< 0.001
BM1s	5	Yes	30	864.810	< 0.001
EB	7	No	21	877.841	< 0.001
OU1	2	No	35	772.582	0.041
BMM	3	Yes	50	841.946	< 0.001
BMMs	4	Yes	60	845.752	< 0.001
**OUM**	**1**	**Yes**	**45**	**766.299**	**0.959**

Description of model parameters and relative goodness of fit. Corrected Akaike Information Criterion (AICc) values and AICc model weights (ωAICc). The model with the highest AICc weight is highlighted in bold. df: degrees of freedom. BM1: single-rate Brownian Motion (BM) model with shared ancestral state. BM1s: single rate BM model with a unique ancestral state for each regime. BMM: multiple-rate BM model with shared ancestral state. BMMs: multiple-rate BM model with a unique ancestral state for each regime. EB: early burst model. OU1: single-optimum Ornstein-Uhlenbeck (OU) model. OUM: multiple-optima OU model.

Supplementary Table 11 | Parameter estimates for the multiple-optima Ornstein-Uhlenbeck model.

Diet/Trait	Parameter	PC1	PC2	PC3	PC4	PC5
Herbivore	θ	0.054	1.244	0.079	-0.320	0.198
Omnivore	θ	-0.476	-0.100	0.069	-0.151	0.054
Predatory	θ	-0.492	-0.101	-0.226	-0.091	0.018
PC1	α	0.009	0.003	-0.006	-0.007	0.004
PC2	α	0.003	0.199	0.010	0.072	-0.036
PC3	α	-0.006	0.010	0.111	-0.046	0.006
PC4	α	0.007	0.072	-0.046	0.079	0.011
PC5	α	0.004	-0.036	0.006	0.011	0.142
PC1	σ	0.113	-0.040	-0.061	0.027	0.030
PC2	σ	-0.040	0.201	-0.011	0.047	-0.023
PC3	σ	-0.061	-0.011	0.058	-0.025	0.003
PC4	σ	0.027	0.047	-0.025	0.024	0.003
PC5	σ	0.030	-0.023	0.003	0.003	0.024

Diet-specific inferred optimum values (θ), strength of selection (α), and drift (σ) estimated with the multi-peak multivariate Ornstein-Uhlenbeck model (OUM).

Supplementary Table 12 | Squamate clades showing rate shifts in trait-independent models of speciation and extinction.

Node	Replicates	Mean speciation rate (My^{-1})	Standard deviation	Ratio to mean outgroup rate	Mean extinction rate (My^{-1})	Standard deviation	Ratio to mean outgroup rate	Cusp number transition	Plant consumption transition
555 (A)	9/10	0.0523	0.0159	4.40	0.0092	0.0030	0.65	-	-
679 (B)	6/10	0.1296	0.0659	2.59	0.0128	0.0083	1.36	-	I → O
691	1/10	0.0511	0.0207	1.01	0.0122	0.0049	1.82	-	-
697 (C)	9/10	0.0875	0.0489	1.72	0.0961	0.0572	11.06	2 → >3	-
705	1/10	0.0523	0.0212	1.03	0.0004	0.0002	0.05	-	-
763	2/10	0.1918	0.0899	3.69	0.1133	0.0553	11.00	2 → 1	-
782 (D)	10/10	0.1439	0.0482	2.86	0.0308	0.0247	3.37	-	I → O
802 (E)	10/10	0.0596	0.0203	1.17	0.0557	0.0189	6.39	-	-
824 (F)	7/10	0.0624	0.0296	1.26	0.0021	0.0014	0.21	-	-
826	1/10	0.0778	0.0315	1.54	0.0060	0.0024	0.49	-	-
846 (G)	10/10	0.3912	0.0966	7.79	0.0433	0.0456	4.69	-	-
914 (H)	10/10	0.1460	0.0231	2.92	0.0143	0.0111	1.55	-	-
996 (I)	5/10	0.0797	0.0417	1.71	0.0100	0.0052	1.15	1 → 3	I → O
1003 (J)	5/10	0.0802	0.0420	1.71	0.0056	0.0030	0.61	-	-
1010 (K)	10/10	0.1586	0.0154	3.16	0.0159	0.0153	1.72	3 → 1	-
1055 (L)	10/10	0.3224	0.0939	6.43	0.0268	0.0181	2.89	-	-
1084 (M)	6/10	0.2555	0.1330	5.15	0.0272	0.0198	3.04	3 → >3	-
1085	4/10	0.2934	0.1555	5.91	0.0347	0.0260	3.63	-	-

Mean rates of speciation and extinction for the 18 clades defined by a rate shift in 10 maximum shift credibility configuration (MSC) independent replicates, including the 13 clades with rate shifts in at least five replicates (A-M, as indicated in Fig. 4c), and their ratio to the corresponding mean rate for the outgroup. Shift location is given by the number of the node immediately above it (see Supplementary Figure 3). Letters in brackets in the "Node" column denote clade labels in Fig. 4c. Column "Replicates" indicates the number of occurrences of a given shift among the ten MSC replicates. Column "Cusp number transition" indicates changes in tooth complexity inferred at a given node, if any. Column "Plant consumption transition" indicates changes in plant matter proportion in the diet inferred at a given node, if any (I: insectivorous, O: omnivorous). A: crown Squamata. B: Egerniinae. C: Polyglyphanodontia. D: *Podarcis*. E: Mosasauria. F: crown Alethinophidia. G: *Chilabothrus*. H: *Varanus*. I: total group Pleurodonta. J: crown Pleurodonta. K: *Phrynosoma*. L: unnamed clade including the most recent common ancestor (MRCA) of *Liolaemus darwinii* and *L. scapularis* and all its descendants. M: unnamed clade including the MRCA of *Ctenosaura quinquecarinata* and *Cyclura cornuta* and all its descendants. Five shifts towards increased speciation coincide exactly with increases in tooth complexity (Polyglyphanodontia (C) and a sub-clade of Iguanidae (M)), plant consumption (Egerniinae (B) and *Podarcis* (D)) or both (total-group Pleurodonta (I)).

Conversely, only two increases in speciation rate coincide with decreases in tooth complexity (total group *Gallotia* (node 763), and genus *Phrynosoma* (K)), and none with decreases in plant consumption.

Supplementary Table 13 | Tooth complexity and plant consumption changes in the vicinity of rate shifts in trait-independent models of speciation and extinction

Node	Cusp number transition (one node below)	Cusp number transition (one node above)	Plant consumption transition (one node below)	Plant consumption transition (one node above)
555 (A)	-	-	-	-
679 (B)	-	-	-	-
691	-	1 → 2	-	-
697 (C)	-	>3 → 3	I → H	H → O
705	-	-	I → H	-
763	-	-	-	-
782 (D)	-	-	-	-
802 (E)	-	-	-	-
824 (F)	-	-	-	-
826	-	-	-	-
846 (G)	-	-	-	-
914 (H)	-	-	-	-
996 (I)	-	-	-	-
1003 (J)	-	-	-	O → I
1010 (K)	-	-	-	-
1055 (L)	-	-	-	O → I
1084 (M)	-	-	O → H	-
1085	3 → >3	-	-	-

All 18 clades defined by a rate shift in 10 maximum shift credibility configuration (MSC) independent replicates, including the 13 clades with rate shifts in at least five replicates (as indicated in Fig. 4c). Shift location is given by the number of the node immediately above it (see Supplementary Figure 3). The columns "Cusp number transition (one node below/above)" indicate changes in tooth complexity inferred in nodes immediately adjacent to a shift location, if any. Similarly, the columns "Plant consumption transition (one node below/above)" indicate changes in plant matter proportion in the diet in nodes immediately adjacent to a shift location, if any (I: insectivorous, O: omnivorous, H: herbivorous). A: crown Squamata. B: Egerniinae. C: Polyglyphanodontia. D: *Podarcis*. E: Mosasauria. F: crown Alethinophidia. G: *Chilabothrus*. H: *Varanus*. I: total group Pleurodonta. J: crown Pleurodonta. K: *Phrynosoma*. L: unnamed clade including the most recent common ancestor (MRCA) of *Liolaemus darwinii* and *L. scapularis* and all its descendants. M: unnamed clade including the MRCA of *Ctenosaura quinquecarinata* and *Cyclura cornuta* and all its descendants. In addition to the five direct correspondences between increased speciation and increases in cusp number/plant consumption (see Supplementary Table 12), Polyglyphanodontia (C), the Iguanidae sub-clade (M), and three more speciation shifts (total group Teiioidea (node 691), crown Teiioidea (node 705), and an unnamed sub-clade of Iguanidae including the MRCA of *Ctenosaura quinquecarinata* and *Iguana iguana* and all its descendants (node 1085) sit within

one node of an increase in tooth complexity or plant consumption. Besides the two correspondences between shifts in speciation rate and decreasing cusp number/plant consumption (see Supplementary Table 12), three other shifts are within one node of such a decrease: Polyglyphanodontia (C), crown Pleurodonta (J), and the Liolaemidae sub-clade (L).

Supplementary Table 14 | Tests of trait-dependent models of squamate diversification.

Model	Hidden states	Character-dependent	Number of diversification rates	Number of transition rates	Dual transitions	AICc (tooth complexity)	ωAICc (tooth complexity)	AICc (diet)	ωAICc (diet)
HiSSE complete, ARD	Yes	Yes	4	12	Yes	5231.30	0.004	5301.83	0.068
HiSSE complete ER	Yes	Yes	4	1	Yes	5275.11	< 0.001	5381.02	< 0.001
**HiSSE no dual ARD**	**Yes**	**Yes**	**4**	**8**	**No**	**5221.05**	**0.595**	**5296.89**	**0.802**
HiSSE no dual 3 rates	Yes	Yes	4	3	No	5247.47	< 0.001	5306.42	0.007
HiSSE no dual ER	Yes	Yes	4	1	No	5271.91	< 0.001	5370.23	< 0.001
BiSSE	No	Yes	2	2	-	5285.85	< 0.001	5362.89	< 0.001
CID-4 3 rates	Yes	No	4	3	No	5221.96	0.378	5300.65	0.123
CID-4 ER	Yes	No	4	1	No	5244.80	< 0.001	5353.53	< 0.001
CID-2 complete ARD	Yes	No	2	12	Yes	5245.97	< 0.001	5362.36	< 0.001
CID-2 complete ER	Yes	No	2	1	Yes	5270.88	< 0.001	5377.14	< 0.001
CID-2 no dual ARD	Yes	No	2	8	No	5239.53	< 0.001	5345.35	< 0.001
CID-2 no dual 3 rates	Yes	No	2	3	No	5227.58	0.023	5310.76	< 0.001
CID-2 no dual ER	Yes	No	2	1	No	5260.71	< 0.001	5364.75	< 0.001

Description of model parameters and relative goodness of fit. For both tooth complexity and plant consumption, a "hidden state" speciation and extinction model (HiSSE) is best supported to account for squamate diversification patterns. Corrected Akaike Information Criterion (AICc) values and AICc model weights (ωAICc) for the binary tooth complexity dataset and the binary diet dataset. The model with the highest AICc weight is highlighted in bold. HiSSE: hidden state speciation and extinction model; BiSSE: binary state speciation and extinction model; CID: character-independent (null) model; "complete": all character state transitions possible, including simultaneous transitions in both the observed and hidden trait; "no dual": simultaneous transitions in both the observed and hidden trait are excluded; ARD: all rates different; ER: equal rates.
