# Supplementary material for: Multiple evolutionary origins and losses of tooth complexity in squamates
Source: Nat Commun. 2021 Oct 14;12:6001. doi: 10.1038/s41467-021-26285-w (PMC8516937; doi:10.1038/s41467-021-26285-w)
Supplement: Supplementary file 3 — Description of Additional Supplementary Files [file 41467_2021_26285_MOESM3_ESM.pdf]

Description of Additional Supplementary Files

File name: Supplementary Data 1

Description: Time-calibrated phylogenetic tree of 545 squamate species and their outgroup, in Newick format.

File name: Supplementary Data 2

Description: Dichotomous time-calibrated phylogenetic tree of 545 squamate species and their outgroup, in Newick format.

File name: Supplementary Data 3

Description: Species-level dataset in CSV format including taxonomic information (columns "species", "order", "suborder", "family"), living/fossil status ("status"), four-state tooth complexity level ("tooth.complexity") with three alternative binarizations ("tooth.complexity.bin1", "tooth.complexity.bin2", "tooth.complexity.bin3"), diet data ("diet") with an alternative three-state coding ("diet3") and two alternative binarizations ("diet.bin1" and "diet.bin2"), references for tooth complexity, including a unique identifier and specimen identification ("reference.teeth", "reference.teeth.id", "specimen"), references for diet data with a unique identifier ("reference.diet", "reference.diet.id"), rate scalars for the variable rates model of tooth complexity and diet ("var.rates.teeth", "var.rates.diet"), speciation and extinction rates averaged over ten independent BAMM replicates ("mean.spe", "mean.ext"), speciation and extinction rates for the best performing tooth complexity- and dietdependent HiSSE model ("hisse.teeth.spe", "hisse.teeth.ext", "hisse.diet.spe", "hisse.diet.ext"), principal component scores ("PC1"-"PC42"), discriminant function scores ("Axis1" "Axis42"), and phylogenetic principal component scores ("pPC1"-"pPC42") for the Discrete Cosine Transform analysis, and rate scalars for the variable rates model of 2D tooth shape evolution ("var.rates.2DGMM").

File name: Supplementary Data 4

Description: Two-dimensional tooth outlines (zipped). File names are formatted for use with Momocs 1.3.0 for R (i.e., "Genus-species_Diet_status.txt").
