# Supplementary material for: Multiple evolutionary origins and losses of tooth complexity in squamates
Source: Nat Commun. 2021 Oct 14;12:6001. doi: 10.1038/s41467-021-26285-w (PMC8516937; doi:10.1038/s41467-021-26285-w)
Supplement: Supplementary file 8 — Reporting Summary [file 41467_2021_26285_MOESM8_ESM.pdf]

Corresponding author(s): Fabien Lafuma, Ian J. Corfe, Nicolas Di-Poï

Last updated by author(s): Sep 15, 2021

Reporting Summary

Nature Portfolio wishes to improve the reproducibility of the work that we publish. This form provides structure for consistency and transparency in reporting. For further information on Nature Portfolio policies, see our Editorial Policies and the Editorial Policy Checklist.

Statistics

For all statistical analyses, confirm that the following items are present in the figure legend, table legend, main text, or Methods section.

n/a	Confirmed	
☐	☒	The exact sample size (*n*) for each experimental group/condition, given as a discrete number and unit of measurement
☒	☐	A statement on whether measurements were taken from distinct samples or whether the same sample was measured repeatedly
☐	☒	The statistical test(s) used AND whether they are one- or two-sided<br>*Only common tests should be described solely by name; describe more complex techniques in the Methods section.*
☒	☐	A description of all covariates tested
☐	☒	A description of any assumptions or corrections, such as tests of normality and adjustment for multiple comparisons
☐	☒	A full description of the statistical parameters including central tendency (e.g. means) or other basic estimates (e.g. regression coefficient) AND variation (e.g. standard deviation) or associated estimates of uncertainty (e.g. confidence intervals)
☐	☒	For null hypothesis testing, the test statistic (e.g. *F*, *t*, *r*) with confidence intervals, effect sizes, degrees of freedom and *P* value noted<br>*Give P values as exact values whenever suitable.*
☐	☒	For Bayesian analysis, information on the choice of priors and Markov chain Monte Carlo settings
☒	☐	For hierarchical and complex designs, identification of the appropriate level for tests and full reporting of outcomes
☐	☒	Estimates of effect sizes (e.g. Cohen's *d*, Pearson's *r*), indicating how they were calculated

Our web collection on statistics for biologists contains articles on many of the points above.

Software and code

Policy information about availability of computer code

Data collection	Surface rendering of CT-scan data was generated with Amira 5.5.0 (Thermo Fisher Scientific, RRID:SCR_007353). We used ImageJ 1.47v (NIH, RRID:SCR_003070) to capture two-dimensional tooth outlines.
Data analysis	We normalised 2D tooth outlines using the EqualSpace function of the package PollyMorphometrics 10.1 (https://pollylab.indiana.edu/software/index.html) for Mathematica 10 (Wolfram Research, RRID:SCR_014448). Published open-source packages (ape 5.3, RRID:SCR_017343; BAMMtools 2.1.6; CODA 0.19-3; ggtree 1.8.1; hisse 1.9.5; Momocs 1.3.0; mvMORPH 1.1.4; phytools 0.6-99, RRID:SCR_015502; rcompanion 2.3.25; viridis 0.5.1, RRID:SCR_016696) for R 3.6.1 (RRID:SCR_001905) were used to analyse data. We used the Markov Chain Monte Carlo (MCMC) and reversible jump Markov Chain Monte Carlo (rjMCMC) algorithms of BayesTraits 3.0.2 (http://www.evolution.rdg.ac.uk/, RRID:SCR_014487) for models of correlated evolution and variable rates models, respectively. We used the rjMCMC algorithm of BAMM 2.6 to generate trait-independent models of speciation and extinction. We used the functions of Mitchell et al. 2018 (https://doi.org/10.5061/dryad.50m70) to generate calibrations and resolve fossil polytomies based on last appearance dates and estimated rates of speciation, extinction, and preservation. All packages and functions were used without modification nor addition of original code.

For manuscripts utilizing custom algorithms or software that are central to the research but not yet described in published literature, software must be made available to editors and reviewers. We strongly encourage code deposition in a community repository (e.g. GitHub). See the Nature Portfolio guidelines for submitting code & software for further information.

Data

Policy information about availability of data

All manuscripts must include a data availability statement. This statement should provide the following information, where applicable:

- Accession codes, unique identifiers, or web links for publicly available datasets
- A description of any restrictions on data availability
- For clinical datasets or third party data, please ensure that the statement adheres to our policy

All datasets generated and analysed during the current study (tip-state dataset, polytomous and dichotomous versions of our phylogeny, 2D outlines) are available as Supplementary Data files 1-4. We used the Reptile Database (http://www.reptile-database.org) to access taxonomic information on extant species and the Paleobiology Database (https://www.paleobiodb.org) for taxonomy and temporal ranges of fossil species. Dietary data were extracted from the database of Meiri (https://doi.org/10.1111/geb.12773) and the published literature (see Supplementary Data 3). CT-scan data are in part publicly available on the Digimorph database (http://digimorph.org/) and the published literature (see Supplementary Data 3). The remnant data are available through N.D.-P., upon reasonable request. Source data are provided with this paper.

Field-specific reporting

Please select the one below that is the best fit for your research. If you are not sure, read the appropriate sections before making your selection.

☐ Life sciences ☐ Behavioural & social sciences ☒ Ecological, evolutionary & environmental sciences

For a reference copy of the document with all sections, see nature.com/documents/nr-reporting-summary-flat.pdf

Ecological, evolutionary & environmental sciences study design

All studies must disclose on these points even when the disclosure is negative.

Study description	Here we study the evolution of tooth complexity and diet in squamates using geometric morphometric and phylogenetic comparative methods. We first statistically test the effect of diet on cusp number. We then perform phylogenetic and non-phylogenetic Principal Component Analyses (PCA) and a Discriminant Function Analysis (DFA) on tooth outlines, and jointly test for phylogenetic signal and morphological differences between diets. Next, we reconstruct tooth complexity and diet ancestral states using maximum likelihood and test for their correlated evolution through a Monte Carlo Markov Chain (MCMC) algorithm. We then use a reversible jump Monte Carlo Markov Chain (rjMCMC) algorithm to detect heterogeneity in tooth complexity and diet transition rates. Subsequently, we detect shifts in the rate of tooth shape evolution with an rjMCMC algorithm and fit different multivariate models of continuous trait evolution (Brownian Motion, Early Burst, and Ornstein-Uhlenbeck) to the tooth shape dataset. To evaluate the role of tooth complexity and diet in squamate macroevolution, we fit and compare trait-dependent diversification models, and statistically test the effect of multiple-cusped teeth and plant consumption on estimated speciation and extinction rates. Furthermore, we compare these results with an rjMCMC fossilized birth-death trait-independent model of diversification and identify shifts in diversification rates, which we compare to tooth complexity and diet ancestral states.
Research sample	The research sample comprises 545 squamate species (428 extant and 117 extinct). Sample size was not predetermined and is meant to representatively cover the whole taxonomic and recorded historic range of Squamata, including the oldest squamate known to date. In addition, two rhynchocephalians (the only extant member of Sphenodontidae and a fossil) and a member of stem-Lepidosauria constitute the outgroup. Fossils were sampled in equivalent proportion from the Mesozoic and Cenozoic records. All 548 species were character-coded for tooth complexity and diet based on data from museum specimens, laboratory animals, and the literature. A subset of 75 species representing the main groups of squamates bearing multiple-cusped teeth was analysed using geometric morphometrics.
Sampling strategy	Sample size was not predetermined and data from specimens and the literature were collected to representatively cover the whole taxonomic and recorded historic range of Squamata. Sampling difficulties precluded access for some particular species due to scarcity of specimen and/or protection status and/or a geographically restricted distribution. Fossils were sampled in equivalent proportion from the Mesozoic and Cenozoic records.<br>Specimens were selected for geometric morphometric analyses to represent all major groups of squamates with multiple-cusped teeth and based on the quality of the material available. We selected left upper posterior maxillary teeth in labial view, choosing whenever possible the tooth with the most numerous cusps in the quadrant. In case no left maxillary tooth was sampled or suitable to trace an outline, we referred to the right quadrant or the lower jaws and mirrored the outline adequately to retain the same orientation.
Data collection	F.L. collected all discrete dental and dietary data from the literature and captured semilandmarks for two-dimensional tooth outlines. Filipe Oliveira Da Silva acquired photographs of specimens from the dry collections of the Museum für Naturkunde (Berlin, Germany). Newly produced CT-scan data was acquired by F.L., Arto Koistinen, Simone Macrì, Kristin Mahlow, and Filipe Oliveira Da Silva.
Timing and spatial scale	Newly produced photographic and CT-scan data were acquired at the Museum für Naturkunde (Berlin, Germany) in March 2014, and CT-scan data were acquired at the University of Eastern Finland (Kuopio, Finland) throughout 2014 and at the University of Helsinki (Finland) from 2014 to 2016. Data collection from the literature and public databases was carried out over the period 2014-2019.
Data exclusions	Taxa were excluded only when tooth complexity, diet, and phylogenetic information were not all available. To control for the effect of aquatic taxa on diversification rates estimated for the Cretaceous Terrestrial Revolution (KTR), we replicated the rjMCMC fit of the trait-independent diversification model with these taxa (ten mosasaurs, three snakes) removed.

Reproducibility	Essential methods and parameters are described in the Methods section. All analysed datasets are available as Supplemetary Data 1-4. Unless specified otherwise, all analyses were replicated at least twice independently (ten independent replicates for the trait-independent diversification model) with consistent results.
Randomization	Permutations and bootstrap were used to assess significance and effect size (see Methods). To avoid over-sampling Liolaemidae, we randomly selected species with complete information (cusp number, diet, phylogeny) according to the relative abundance of dietary categories within the group and of liolaemids among squamates.
Blinding	Species were sampled to cover the whole temporal and taxonomic extent of Squamata and regrouped by diet categories for analysis, making blinding not relevant to the study.

Did the study involve field work? ☐ Yes ☒ No

Reporting for specific materials, systems and methods

We require information from authors about some types of materials, experimental systems and methods used in many studies. Here, indicate whether each material, system or method listed is relevant to your study. If you are not sure if a list item applies to your research, read the appropriate section before selecting a response.

Materials & experimental systems

n/a	Involved in the study
☒	☐ Antibodies
☒	☐ Eukaryotic cell lines
☐	☒ Palaeontology and archaeology
☐	☒ Animals and other organisms
☒	☐ Human research participants
☒	☐ Clinical data
☒	☐ Dual use research of concern

Methods

n/a	Involved in the study
☒	☐ ChIP-seq
☒	☐ Flow cytometry
☒	☐ MRI-based neuroimaging

Palaeontology and Archaeology

Specimen provenance	We collected data on fossil specimens only from published literature.
Specimen deposition	All specimens referred are deposited in museums and international institutions. See Supplementary Data 3 and associated references for full information on specimen repositories.
Dating methods	No new fossil calibrations are proposed.

☐ Tick this box to confirm that the raw and calibrated dates are available in the paper or in Supplementary Information.

Ethics oversight	All fossil data were accessed through published literature, and thus no ethical approval was required.

Note that full information on the approval of the study protocol must also be provided in the manuscript.

Animals and other organisms

Policy information about studies involving animals; ARRIVE guidelines recommended for reporting animal research

Laboratory animals	Five squamate species (Ameiva amevia, Anolis equestris, Eublepharis macularius, Oplurus cyclurus, Varanus acanthurus) were represented by one (sub)adult (8 to 18 months post-hatching) of undetermined sex acquired from specialised retailers (see Supplementary Data 3).
Wild animals	The study did not involve wild animals.
Field-collected samples	The study did not involve samples collected in the field.
Ethics oversight	The Laboratory Animal Center (LAC) of the University of Helsinki and/or the National Animal Experiment Board (ELLA) in Finland approved all reptile captive breeding (license numbers ESLH-2007-07445/ym-23 and ESAVI/7484/04.10.07/2016).

Note that full information on the approval of the study protocol must also be provided in the manuscript.
